# Microstructure Evolution in He-Implanted Si at 600 °C Followed by 1000 °C Annealing

**DOI:** 10.3390/ma14175107

**Published:** 2021-09-06

**Authors:** Zhen Yang, Zhiping Zou, Zeyang Zhang, Yubo Xing, Tao Wang

**Affiliations:** 1 Sino-French Institute of Nuclear Engineering and Technology, Sun Yat-sen University, Zhuhai 519082, China; zouzhp6@mail2.sysu.edu.cn (Z.Z.); zhangzy228@mail2.sysu.edu.cn (Z.Z.); xingyb3@mail2.sysu.edu.cn (Y.X.); 2 Institute of Fluid Physics, China Academy of Engineering Physics, Mianyang 621900, China

**Keywords:** microstructure, cavities, he implantation, annealing

## Abstract

Si single crystal was implanted with 230 keV He^+^ ions to a fluence of 5 × 10^16^/cm^2^ at 600 °C. The structural defects in Si implanted with He at 600 °C and then annealed at 1000 °C were investigated by transmission electron microscopy (TEM) and high-resolution transmission electron microscopy (HRTEM). The microstructure of an as-implanted sample is provided for comparison. After annealing, rod-like defects were diminished, while tangled dislocations and large dislocation loops appeared. Dislocation lines trapped by cavities were directly observed. The cavities remained stable except for a transition of shape, from octahedron to tetrakaidecahedron. Stacking-fault tetrahedrons were found simultaneously. Cavity growth was independent of dislocations. The evolution of observed lattice defects is discussed.

## 1. Introduction

In the last decade, light ion implantation has been used particularly in the microelectronics field for manufacturing advanced electronic devices on silicon-on-insulator (SOI) substrate, which can be produced by two different kinds of methods [1,2,3,4]. One is high dose oxygen implantation, followed by under 1350 °C annealing for 1 to 2 h. The other is based “smart-cut” technology, which was firstly reported by Bruel [1]. The procedure of this method comprises hydrogen or hydrogen/helium co-implantation into silicon, bonding them to a substrate stiffener and then annealing at a low temperature for crack growth. The schematic flow chart for the synthesis process can be found in the literature [5]. In detail, the procedure of the method starts with high-dose H implantation [6,7]. H+ ions are implanted into a Si substrate, and during annealing, the hydrogen atoms and some of the vacancies generated by implantation precipitate and form platelets [8,9]. These platelets, filled with H2 gas, grow in size during annealing until they become large enough and elastically interact, before finally coalescing to form micro-cracks distributed within a thin layer at a certain depth from the wafer surface [9,10,11]. When these micro-cracks are close to the free surface of the wafer, the stress generated in the semiconductor matrix by the pressure inside the micro-cracks can elastically relax through the deformation of the surface, i.e., the formation of blisters [7,12]. In order to decrease the implantation dose, H+ and He+ ions are used; helium incorporates and over-pressurizes the hydrogen platelets during annealing and thus promotes their more effective mechanical coalescence and the formation of blisters [13,14]. The smart-cut process depends on different parameters, such as temperature and annealing time [8], He and H fluences [14], the He to H fluences ratio [15], He and H relative depth distributions (imposed by respective ion energies [16]) and the relative order of He and H implantation [17]. In the early period, most interest in the applications of this method was devoted to studying helium bubble formation and evolution in semiconductors [18,19,20,21,22,23]. During He implantation, numerous helium atoms and cascade collision-induced Frenkel pairs are introduced. Due to the low solubility of He in Si, He atoms are inclined to interact with vacancies to form He-V complexes. After thermal annealing at low temperatures (even at room temperature), He-V complexes tend to migrate and agglomerate into He bubbles. These bubbles exhibit a spherical shape and have a very high inner pressure, which can be released by emitting Si interstitials (i.e., the dislocation loop punching phenomenon) [18]. Frequently, {113} defects are found around bubbles [19]. At high temperatures (700 °C and above), He atoms can escape from the bubbles, leaving cavities embedded inside the crystalline silicon [20]. There are four kinds of potential applications for cavities in silicon: (1) Cavities offer the efficient gettering of transition metals (i.e., Au, Pt and Cu) due to dangling bonds on the void inner surfaces [24]. (2) Cavities introduce deep levels in the silicon band gap that can affect the charge-carrier lifetime, to locally control the lifetime of carriers [25]. (3) Cavities could suppress the formation of extended defects which act as the getters for Si interstitials and thus can control the dopant diffusivity [2]. (4) Surface wafer exfoliation can be facilitated via cavities, in order to fabricate SOI structures [26].

Griffioen et al. [27] first reported He bubble formation in He-implanted Si. Because the vacancy–helium atom interaction is repulsive, helium atoms easily escape from the silicon wafer at low temperatures. Contrary to the case of He-implanted metals or SiC [28,29,30], He bubble formation depends on the He concentration in a local area. Raineir et al. [31] argued that the threshold dose for the formation of He bubbles is (3.5 ± 1) × 10^20^/cm^3^ at room temperature, corresponding to an implanted fluence as low as 1 × 10^16^/cm^2^. David et al. [32] investigated the formation of He bubbles and extended defects in He-implanted Si to a fluence of 5 × 10^16^/cm^2^ at a temperature range from room temperature to 800 °C. At room temperature and 300 °C, bubbles with a mean size of 2–3 nm and a density of (4 ± 1) × 10^16^/cm^3^ were formed. An increase in size but a decrease in the density of bubbles was noticed at 600 °C. Meanwhile, ion implantation can introduce large supersaturations of extended defects, i.e., elongated rod-like defects and large ribbon-like defects. No cavities were observed at 800 °C, except for dislocation loops. Han et al. [19] recently reported 230 keV He-implanted Si at 600 °C, where many rod-like defects belonging to {113}, {111} and ($\bar{2}00$) were formed at the tail of the damaged band. In the front of the damaged layer, many ribbon-like defects were observed. Extended defects interact with the dopants, causing transient-enhanced diffusion (TED) [33,34]. TED can lead to a change in the junction depth and increasing leakage current. Therefore, it is worth understanding the diffusion processes of both intrinsic point defects and dopants, to satisfy demand for future-generation electronic devices. In order to reduce residual extended defects, three different kinds of methods can be used. The first is He implantation at an elevated temperature due to the increase in dynamic annealing [19]. The second is thermal annealing [31]. The third is damage recovery via swift heavy-ion irradiation [35]. It has been widely reported that electronic excitation promotes recrystallization by influencing the migration of pre-existing defects in Si and SiC [36,37]. The enhanced growth of He bubbles and an increase in the size of the lattice defects was found previously after Ar ion irradiation at an energy of 792 MeV at room temperature. According to much previous literature, the cavities remain stable and few extended defects survive thermal annealing at 1000 °C and above. Therefore, thermal annealing is a good candidate and much more efficient than implantation at high temperatures or swift heavy-ion irradiation. In order to reduce residual defects during the ion implantation, the increase in dynamic annealing via elevated temperature is a good choice. Currently, there are few reports of defect evolution in He-implanted Si at above room temperature followed by high-temperature annealing. The defect microstructure and the interaction between cavities and interstitial-type defects after annealing need further investigation.

In the present study, Si was implanted with 230 keV He ions to a fluence of 5 × 10^16^/cm^2^ at 600 °C and then thermally annealed at 1000 °C for 30 min. In order to investigate the influence of thermal annealing on microstructure evolution, an as-implanted sample was analyzed by conventional transmission electron microscopy (TEM). The microstructure of tangled dislocations, Frank loops, stacking-fault tetrahedrons and cavities was characterized by high-resolution transmission electron microscopy (HRTEM). The results not only give a deep understanding of how to control the size, distribution and arrangement of both cavities and extended defects, but also provide insight into the application of light ion implantation for the development of microelectronic devices.

## 2. Experimental Process

A Czochralski-grown (Cz–Si) n-type (100) Si wafer, with a resistivity of 0.6–0.79 Ω∙cm, was implanted with 230 keV He^+^ ions to a fluence of 5 × 10^16^/cm^2^ at 600 °C. The beam current density was kept at 1.2 μA/cm^2^. According to a Monte-Carlo code SRIM2008 [38], the implantation doses correspond to a peak damage of 2.2 displacements per atom (dpa), and the peak helium concentration is 3.5 at.% (using a displacement energy of Si = 15 eV and a density of 2.31 g cm^−3^). The implantation experiment was carried out in the 320 kV Multi-discipline Research Platform for Highly Charged Ions of the Institute of Modern Physics, Chinese Academy of Science (CAS). In order to provide an uniform ion fluence across the sample, the beam was rastered by an electrostatic scanner with fixed frequencies of 993 and 990 Hz in horizontal and vertical directions, respectively. The He-implanted sample was annealed at 1000 °C for 30 min in a vacuum environment (≤10^−3^ Pa). The microstructures of the samples before and after annealing were characterized by cross-sectional transmission electron microscopy (XTEM) using a FEI Tecnai G20 (FEI Company, Hillsboro, OR, USA) operated at 200 kV. The samples were observed near the [011] zone axis. A double-tilt goniometer stage was used, in order to tilt the TEM sample to satisfy different diffraction vectors. The micrographic conditions were bright field (BF) and weak-beam dark field (WBDF) with (g, 3g), g = ($11\bar{1}$) near z = [011], where *g* is the diffraction vector and *z* is the zone axis. The XTEM sample was prepared by mechanical thinning and then Ar-ion milling; a detailed illustration is given in Ref. [19].

## 3. Results and Discussion

**Figure 1:**
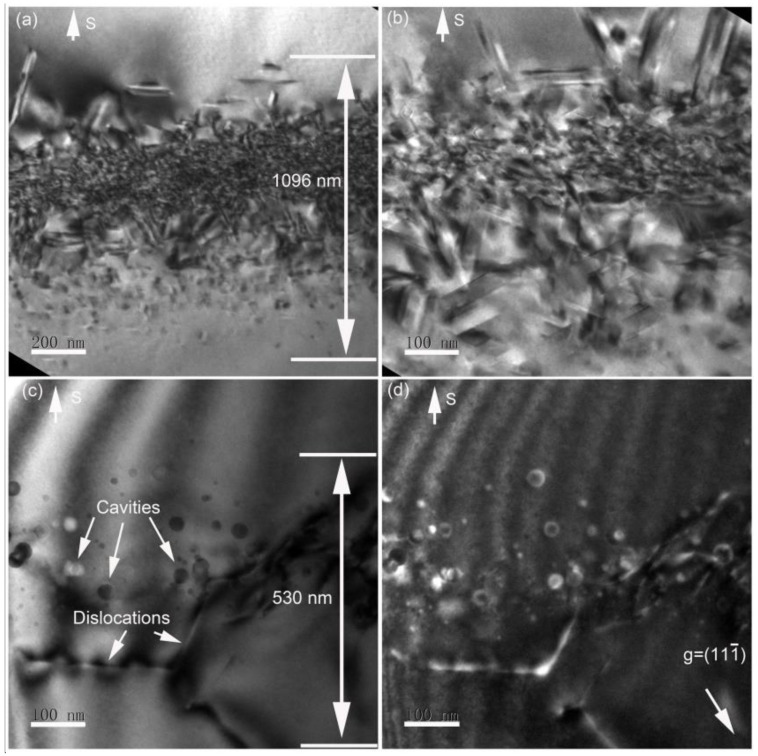
XTEM bright-field micrographs of 230 keV He-implanted Si to a fluence of 5 × 10^16^/cm^2^ at 600 °C. (**a**) Low magnification and (**b**) high magnification after 1000 °C annealing for 30 min, (**c**) bright field and (**d**) weak-beam dark field with g = ($11\bar{1}$). Cavities and dislocations are noted in (**c**). S: surface direction.

**Figure 2:**
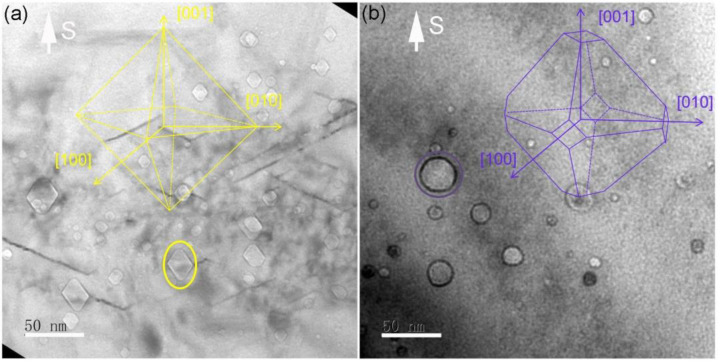
XTEM image under an under-focused condition showing cavities in He-implanted Si at 600 °C. (**a**) As-implanted, (**b**) after 1000 °C annealing. Insets show schematic of the cavity shape consisting of {111} planes, octahedron in (**a**) and tetrakaidecahedron in (**b**).

The defect distribution in the as-implanted and annealed sample are shown in Figure 1. Many lattice defects exhibiting strong diffraction contrasts can be observed in the as-implanted sample (see Figure 1a,b). The damaged layer has a width of approximately 1100 nm. After 1000 °C annealing, an obviously change in the damaged layer appeared. The width of the damaged layer decreased to 530 nm in detail (see Figure 1c), which is half the value of the as-implanted sample. Moreover, the rod defects and ribbon-like defects were inexistent; instead, tangled dislocations and several Frank loops were observed, as shown in Figure 1c,d. Tangled dislocations exhibiting white contrasts shown in Figure 1d had a length of over 200 nm. Some cavities were clearly visible. A comparison of cavity change is presented in Figure 2.

In the as-implanted sample, many octahedron-shaped cavities were observed, as shown in Figure 2a. However, after 1000 °C annealing, some tetrakaidecahedron-shaped cavities were found and the octahedron-shaped cavities were missing, as shown in Figure 2b. Furthermore, the number and density of the observed cavities decreased significantly, because many small cavities seen in Figure 2a disappeared after annealing. In addition, the maximum size of these cavities was not increased compared to the as-implanted sample.

**Figure 3:**
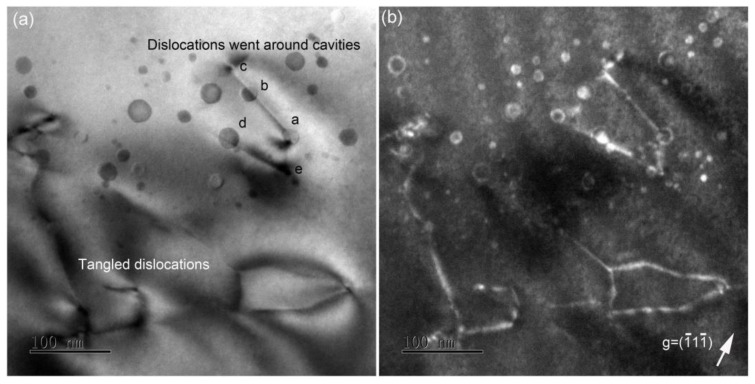
XTEM images showing tangled dislocations and cavities in He-implanted Si at 600 °C followed by 1000 °C annealing. (**a**) Bright field and (**b**) weak-beam dark field with g = ($\bar{1}1\bar{1}$). Dislocations going around cavity *b* or *d* can be seen in (**a**). The direction of the sample surface is up.

In order to investigate whether cavities prefer to nucleate on dislocations during annealing, Figure 3 shows the distribution of dislocations, tangled dislocations and cavities in the damaged layer. It can be seen that tangled dislocations were observed only behind the cavities. Interestingly, no dislocations were found in front of the cavities. On the contrary, many lattice defects were observed at both sides of the cavity layer in the as-implanted sample. A possible reason for this is that the dissociation of dislocations led to interstitials trapped by the sample surface during 1000 °C annealing. Cavities were exhibited circular shape and dark contrasts were observed randomly distributed. Furthermore, some dislocations went around the cavities. As shown in Figure 3a, one dislocation started at cavity *a*, went around cavity *b* and ended at cavity *c*.

**Figure 4:**
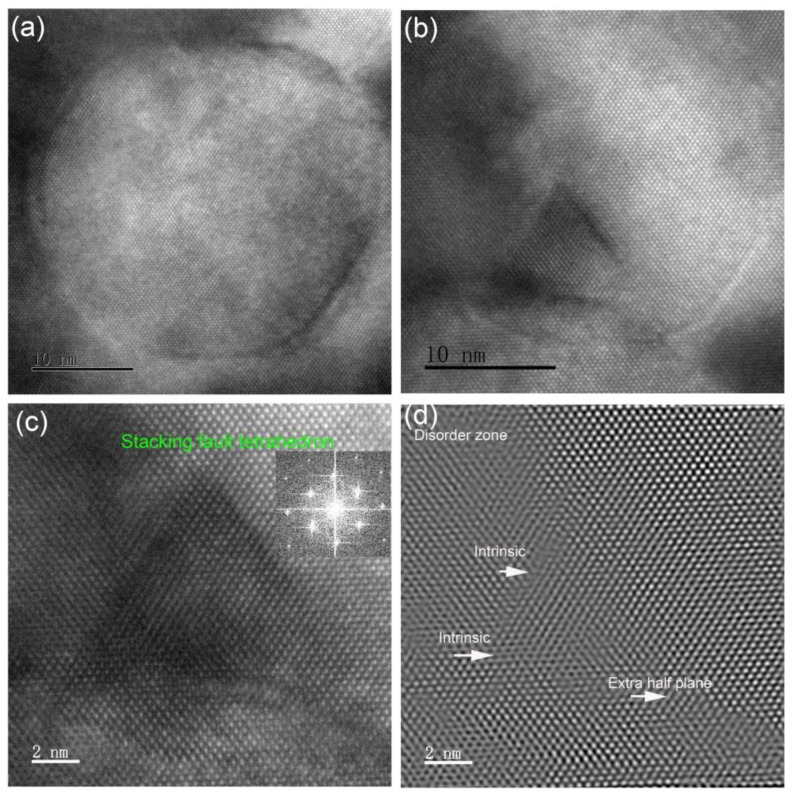
High-resolution TEM images of He-implanted Si at 600 °C followed by 1000 °C annealing showing (**a**) a bubble, (**b**) a stacking-fault tetrahedron above a bubble, (**c**) a stacking-fault tetrahedron only and (**d**) inverse Fourier filtered image of (**c**). Inset shows fast Fourier transform image taken from overhead of the stacking-fault tetrahedron in (**c**).

Figure 4 presents HRTEM micrographs of cavities and stacking-fault tetrahedrons appearing as dark triangles. Cavities have facetted planes, including {111} and {100} planes. The illustration of cavity shape presented in Figure 2b shows that tetrakaidecahedron-shaped cavities formed after 1000 °C annealing. Stacking-fault tetrahedrons were not found in the as-implanted sample. After annealing, these defects were distributed in two different zones, where some stacking-fault tetrahedrons are located above cavities, and the others are in the matrix. The stacking-fault tetrahedrons are composed of vacancies, which can migrate quickly during 1000 °C annealing, but not 600 °C implantation. The electron-diffraction pattern shows symmetrical distribution, unlike Frank loops, which introduce rel-rod streak on {111} planes. This result indicates that the stacking-fault tetrahedrons induce a displacement field in their surroundings that is smaller than the one induced by a Frank loop. It is well recognized that the Burger’s vector of stacking-fault tetrahedron is 1/6<110>, while it is 1/3<111> for a Frank loop. Stacking-fault tetrahedrons are usually found in ion-implanted fcc crystals, like Cu, Ag, Ni [39,40]. Figure 4d confirms many intrinsic defects along the edge of a stacking-fault tetrahedron, and that a highly disordered zone and extrinsic defects formed around it. These lattice defects can produce local strain, which can be characterized by geometric phase analysis (GPA) [41].

**Figure 5:**
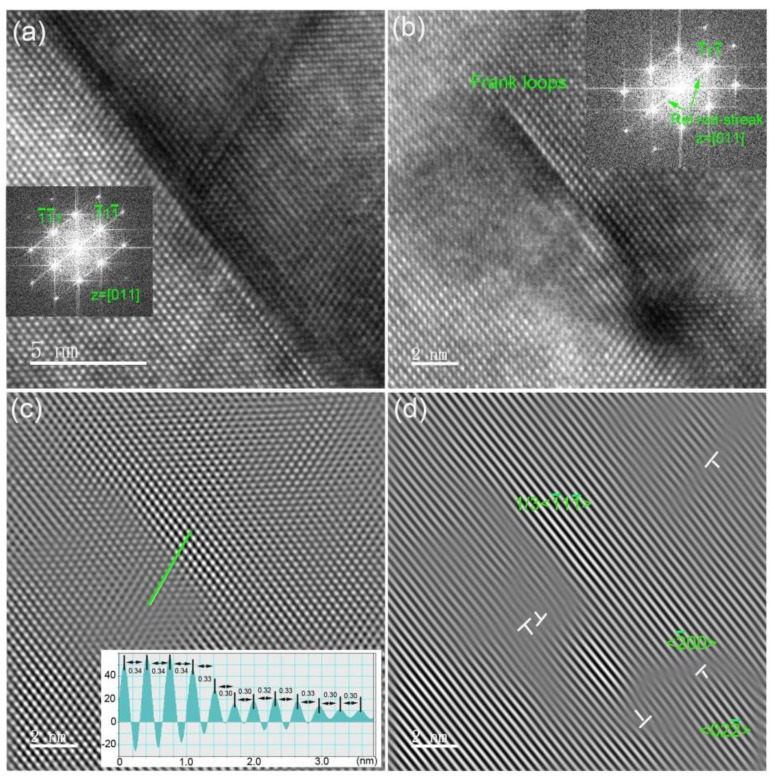
High-resolution TEM image of He-implanted Si at 600 °C followed by 1000 °C annealing showing (**a**) a tangled dislocation, (**b**) a Frank loop, (**c**) inverse Fourier filtered image and profile of signal intensity of the analyzed atoms as indicated by one line across the Frank loop, (**d**) inverse Fourier filter of ($\bar{1}1\bar{1}$) where interstitial-type dislocation loops can be observed. Insets show fast Fourier transform images where rel-rod streaks on {111} planes are visible.

Figure 5 presents the microstructure of a tangled dislocation and a Frank loop. It can be seen that the tangled dislocation is over 20 nm length and 4–5 lattice atom layers. However, the Frank loop is usually less than 10 nm in length, and has one lattice atom layer width. Rel-rod streaks on {111} planes were found in the selected area’s electron-diffraction pattern, indicating tangled dislocations and Frank loops on the {111} plane. The atomic distribution of the Frank loop was analyzed and the result is presented in Figure 5c. It can be seen that the signal intensity of atoms is different in the whole micrograph. No lattice disorder atoms are bright. In addition, the intervals of these atoms can be clearly distinguished, as shown in the inset in Figure 5c. In the disordered zone, the contrast of lattice atoms is dim and the interval between two atoms becomes fuzzy. For example, it is 0.34 nm for two order atoms and decreases to 0.30 nm for two disorder atoms, indicating a decrease in the {111} lattice atom interval to 11.8%. This corresponds to a lattice contraction of 9.6%. The lattice atoms suffered from compressive stress that originated from interstitial-type defects, as shown in Figure 5d. Similarly, Haynes et al. [42] found compressive stress induced in 0.5 keV He-implanted Si at 450 °C. In addition, ($\bar{1}1\bar{1}$) lattice atoms glide, to produce <$\bar{2}00$> and <$02\bar{2}$> type loops, as shown in Figure 5d.

As mentioned by Reineri et al. [31], the formation of a cavity with radius *R_v_* leads to an increase in crystal free energy of *4πR_v_*^2^*σ*, where *σ* is the surface energy density of Si. The value of *V* (*V* stands for vacancy) contained in a cavity is 4*πR_v_*^3^/(3*Ω*) (*Ω* is the volume of one vacancy). At the *V* concentration per unit volume, *C_v_*, the chemical potential of a vacancy is *k_b_T*ln(*C_v_/C_v_^eq^*), where *k_B_* is Boltzmann’s constant, *T* is the absolute temperature, and *C_v_^eq^* is the *V* thermal equilibrium concentration. Therefore, the crystal free energy due to *V* consumption can be expressed as

(1) $$\left(\frac{4\pi R_{V}^{3}}{3\Omega })k_{B}Tln(\frac{C_{V}}{C_{V}^{eq}}\right)$$

Thus, the net Si crystal free energy change is

(2) $$∆G_{V}=4\pi R_{V}^{2}\sigma -(\frac{4\pi R_{V}^{3}}{3\Omega })k_{B}Tln(\frac{C_{V}}{C_{V}^{eq}})$$

At the maximum value the condition where *R_v_* = R_v_^*^,

(3) $$\frac{d\left(∆G_{V}\right)}{dR_{V}}=0$$

yields

(4) $$R_{V}^{*}=\frac{2\sigma \Omega }{k_{B}Tln\left(\frac{C_{V}}{C_{V}^{eq}}\right)}$$

The thermal equilibrium concentration *C_V_^eq^* of vacancies is expressed as

(5) $$C_{V}^{eq}=N\times e^{-\frac{E_{V}}{Tk_{B}}}$$

where *N* is the density of silicon (5 × 10^22^ at/cm^3^). The *V* clusters coalesce into cavities only if their dimension overcomes the critical radius R_v_^*^, otherwise they disappear, corresponding to the Ostwald ripening mechanism.

In the present study, many ribbon-like defects formed in the as-implanted sample have been completely annealed out after 1000 °C annealing. The crystal free energy tends to decrease with increasing temperature. When a cavity of radius *R_v_* is formed, the increase in crystal free energy is expressed as:

Δ*G_V_ = 4πR_V_^2^σ* (6)

where *σ* is the Si surface energy density. For a loop of radius *R_d_* that may or may not comprise an intrinsic stacking fault is given by *πR_d_*2*γ* + 2π*R_d_*(*Γ/L*), where *γ* is the intrinsic stacking-fault energy density, Γ/*L* is the edge dislocation elastic and core energy per unit length:

(7) $$\frac{\Gamma }{L}=\frac{\mu b^{2}}{4\pi \left(1-\nu \right)}\left(ln\frac{8\alpha R_{d}}{b}-1\right)$$

where *μ* is the shear modulus of Si, *b* is the magnitude of the dislocation’s Burgers vector, *ν* is Poisson’s ratio, and *α* is a constant. The crystal free energy increases when a dislocation loop is formed. On the contrary, it will decrease once some vacancies combine with interstitials. The vacancy number for forming the loop is *πR_d_2b/Ω*, where *Ω* is the volume of one vacancy. Therefore, the crystal free energy decreases due to the consumption of vacancies by *πR_d_2b/*Ω × *k_B_T × ln (C_v_/C_v_^eq^).* The net Si crystal free energy change can be expressed as

(8) $$∆G_{d}=\pi R_{d}^{2}\gamma +R_{d}\frac{\mu b^{2}}{2\left(1-\nu \right)}\left(ln\frac{8\alpha R_{d}}{b}-1\right)-\frac{\pi bR_{d}^{2}}{\Omega }k_{B}Tln\frac{C_{V}}{C_{V}^{eq}}$$

According to Raineri et al.’s report, the energy of the defects is defined by Equations (2) and (8) at the equilibrium condition *C_v_* = *C_v_*^eq^, where it is assumed *Ω* = 2 × 20^−23^ cm^3^, *σ* = 1230 ergs cm^−2^, *γ* = 60 ergs cm^−2^ for the faulted dislocation loop, *μ* = 6.46 × 10^11^ dyne cm^−2^, *ν* = 0.228, *α* = 4, *b* = 3.135 × 10^−8^ cm for the 1/3<111> Frank loop and 3.84 × 10^−8^ cm for the 1/2<110> perfect loop. Defect energy increases linearly with vacancy number. Cavities are stable when they contain fewer than 4 × 10^7^ vacancies (≤50 nm in diameter). The present result shows any cavities or stacking-fault tetrahedrons that were less than 50 nm along the long axis.

In Li et al.’s report [43], a high density of extended defects was kept stable in Ar-implanted Si followed by 1100 °C annealing. This can be attributed to cavities that act as a sink for interstitials, and therefore the defect annealing is more efficient in He-implanted Si than Ar implantation. Stolk et al. previously reported that these ribbon-like defects belong to the {311} type, which will dissolve in a temperature range of 670–815 °C [44]. Roqueta et al. [45] argued that the self-interstitials produced by the dissolution of {311} defects can be captured by small cavities, consistent with our finding that many small cavities disappeared after annealing. Interestingly, Figure 3a presents one dislocation that started from cavity *a*, went around cavity *b* and ended at cavity *c*. We regarded that the dislocation went around cavity *b*, not through it, because the dislocation would have been trapped if it went through cavity *b*. In front of the cavity layer, no dislocations were observed. Only a few tangled dislocations behind the cavity layer were formed by the growth of Frank loops. The formation of well-defined facets of cavities is widely reported, whether in semiconductors or metals. This has been determined by the relative free energies of crystallographic planes in a Wulff construction. Eaglesham et al. [46] argued the surface energy of {111} is the lowest, next to {100}, and thus the facets with the lowest surface energy {111} planes will be preferred, i.e., octahedron-shaped cavities formed in the as-implanted sample (see Figure 2a). With increasing temperature, an octahedral shape turns into a truncated octahedron with both {111} and {100} planes. Frank loops and tangled dislocations tend to grow on {111} planes. Around Frank loops, lattice contraction occurs due to the agglomeration of interstitials. These extended defects and lattice strain affect electron transport, and therefore the material needs a higher annealing temperature to recover from these defects [10,47,48].

## 4. Conclusions

The influence of thermal annealing on defect evolution in He-implanted Si at 600 °C was investigated. After 1000 °C annealing, the width of the damaged layer decreased sharply. No dislocations were found in front of the cavity layer; instead, only few tangled dislocations formed behind it. The number of cavities decreased significantly, and most small-sized cavities disappeared. This is attributed to the dissociation of {311} defects, resulting in forming many free interstitials that recombine with cavities. Cavities with a tetrakaidecahedron shape were formed due to the surface energies of different crystallographic planes. Stacking-fault tetrahedrons with a low lattice strain were observed. Around them, Frank loops and a lattice contraction of 9.6% were obtained. A higher temperature would be needed to obtain the complete annealing of the observed Frank loops.

## Data Availability

Not applicable.
